# OLDER ADULTS' WORK DISRUPTIONS IN APRIL/MAY 2020: IMPLICATIONS FOR WORK STATUS AND MENTAL HEALTH OVER 6 MONTHS

**DOI:** 10.1093/geroni/igac059.1190

**Published:** 2022-12-20

**Authors:** Leah Abrams, Jessica Finlay, Lindsay Kobayashi

**Affiliations:** Tufts University, Department of Community Health, Medford, Massachusetts, United States; University of Michigan, Ann Arbor, Michigan, United States; University of Michigan, Ann Arbor, Michigan, United States

## Abstract

Using the COVID-19 Coping Study, we sought to determine how work disruptions for older adults in April/May 2020 related to labor force status in September/October 2020 and mental health throughout those six months (N=2,367). One-third of respondents who lost their job in April/May were unemployed at the end of follow-up, while 15% were unemployed after furloughs and 9% after reduced hours/income. One-quarter of those furloughed in April/May were out of the labor force at follow-up – evidence of a potential pathway from furloughs into retirement. Being employed at follow-up was most common after work-from-home in April/May (90%). Multi-level models revealed differences in mental health trajectories over six months according to baseline work disruptions, including persistently high anxiety following job loss and delayed upticks in anxiety and depressive symptoms when working from home. This research provides insights into longer-term economic and mental health ramifications of pandemic-related work disruptions among older workers.

